# Comparison of under-five mortality for 2000, 2005 and 2011 surveys in Ethiopia

**DOI:** 10.1186/s12889-016-3601-0

**Published:** 2016-09-05

**Authors:** Dawit G. Ayele, Temesgen T. Zewotir

**Affiliations:** 1 Department of Epidemiology, Room E7133, The Johns Hopkins University, Bloomberg School of Public Health, 615 North Wolfe Street, Baltimore, 21205 MD USA; 2 School of Mathematics, Statistics and Computer Science, University of KwaZulu-Natal, Pietermaritzburg, Private Bag X01, Scottsville, 3209 South Africa

**Keywords:** Baseline hazard function, Cross-sectional study, Demographic Health Survey (DHS), Hazard ratio, Survival function

## Abstract

**Background:**

Though the socio-economic situation of the Ethiopian household is improving along with the decrease in under-five child mortality. But, under-five mortality is still one of the major problems. Identification of the risk factors change over time which mismatches with the diminishing rate of under-five mortality is important to address the problems.

**Methods:**

The survey data used for this research was taken from three different Ethiopian Demographic and Health Surveys (2000, 2005 and 2011). This data was used to identify the effect of time varying under-five mortality risk factors. The Cox proportional hazard model was adapted for the analysis.

**Results:**

The effect of respondent’s current age, age at first birth and educational level on the under-five mortality rate significantly diminishes in the recent surveys. On the other hand, the effect of the number of births in the last 5 years increases more in 2011 than in the earlier two surveys. Similarly, number of household members in the house and the number of under-five children in the house demonstrated a difference through years. Regarding total children ever born, child death is more for the year 2000 followed by 2005 and 2011.

**Conclusion:**

Based on the study, our findings confirmed that under-five mortality is a serious problem in the country. The analysis displayed that the hazard of under-five mortality has a decreasing pattern in years. The result for regions showed that there was an increase in years for some of the regions. This research work gives necessary information to device improved teaching for family planning and children health care to change the child mortality circumstance in the country. Our study suggests that the impact of demographic characteristics and socio-economic factors on child mortality should account for their integral changes over time.

**Electronic supplementary material:**

The online version of this article (doi:10.1186/s12889-016-3601-0) contains supplementary material, which is available to authorized users.

## Background

The highest under-five mortality rate is found in Sub-Saharan countries when compared to other countries. This rate is about 7 times greater than WHO European Region [1, 2]. Even though various actions have been taken to decrease under-five mortality, most of the Sub-Saharan countries show very high under-five mortality rates. The rate is above 100 deaths per 1000 live births [3].

It is believed that child mortality rates have a considerable decrease in Ethiopia due to the progressive and consistent implementation of the Health interventions since 1960 [4]. Despite this implementation, under-five mortality rates show the highest trend in Ethiopia [5–7]. Furthermore, crude death rates have also significantly decreased over the past 10 years [8–10]. Based on available information, infant and under-five mortality have constant decrease over the past 25 years. In the last 10 years infant and under-five mortality showed a more pronounced reduction [11, 12].

In fact since 1990 progress in under-five mortality has been made in every region of the world [13]. Despite progress all over the world, the geographic divergence of child mortality continues, and parallels the prevalence of extreme poverty; the sub-Saharan countries have a high rate of births which is above 100 per 1000 live births [13]. Even though it is challenging to identify contributing relations between strategy, programme inputs and health impact, there are credible standards to recognise key policy and programme inputs. One of the reasons could be the implemented strategy to reduce under-five mortality [14] changes in the last 20 years. Nevertheless, such inferences emanate from pieces of cross-sectional studies but not from the longitudinal study.

Likewise, we are aware that there are many cross-sectional studies on under-five mortality in Ethiopia and other countries [15–17]. These cross-sectional studies are incomplete in their capability to draw valid recommendations about any relationship or potential causality over time. Even then no comprehensive analysis over a definite region to include for global and/or local socioeconomic differences on the child mortality was conducted. In 2013, Negera et al. explained trends and differentials of infant and under-five mortality in Ethiopia. But this study mostly presented the descriptive analysis for the three EDHS surveys. The multivariate analysis which is presented in this research paper only shows the overall effects of child mortality for years 2000, 2005 and 2011. This study lacks the inclusion of the time effect in the investigation. Hence, this study attempts to give child mortality model with the reducing effect over time which is appropriate to find the effect of related socio-economic and demographic factors and covariates. Alternatively, the socio-economic and demographic factors can be classified as the mother’s characteristics (such as current age, age at 1st birth, highest educational level, number of under-five children, number of children ever born, number of live births in the last 5 years, current pregnancy status and religion), child specific characteristics (types of birth, gender of the child) and household characteristics (region, place of residence, family size) and environmental characteristics (toilet facility, fuel type, floor material, roof material and wall material) .

The under-five mortality pattern is known to have a decreasing trend. Bearing this in mind fitting a survival analysis with the lifetime distributions of various investigated groups [16, 18] is one of the main problems in the study of life data. In most cases, the impact of different covariate variables on the lifetime as the dependent variable are modelled using regression models. Therefore, these models are an important part of survival analysis [19, 20]. This kind of model is known as the Cox regression model or the proportional hazards regression model [21]. Therefore, this study adapts the Cox proportional hazard model to assess the effect of socio-economic and demographic factors on the decreasing trend of child mortality over time.

## Methods

Ethiopia is located in Eastern Africa which is classified as Sub-Saharan Africa. In 2000, Ethiopia conducted the first Ethiopian Demographic and Health Survey (EDHS). As a continuous study, EDHSs were conducted in 2005 and 2011. This study is a cross-sectional investigation administered at the household level periodically. These surveys consist of 540 to 624 selected enumeration areas (EA). The household listing was performed for each of selected EAs. 2000, 2005 and 2011 EDHS samples were designed to provide estimates for the health and demographic variables of interest for Ethiopia as a whole; urban and rural areas of Ethiopia and 11 geographical areas. For the survey, 14,642 households in 2000, 14,500 in 2005 and 17,817 households in 2011 were included in data collection. For 2000 and 2005, the 1994 Population and Housing Census and for 2011, the 2007 Population and Housing Census frames were used as the sampling frame [8–10]. Different socio-economic, demographic and geographic factors were examined in this study.

2000, 2005 and 2011 EDHS sample was considered to make available estimates for the health and demographic variables at country level and for the urban and rural area of Ethiopia and all the 11 administrative provinces, called regions. The outcome of interest for this study is child death before reaching age five. According to the Millennium Development Goal (MDG), one of the goals is to reduce under-five mortality. Questions related to the advances to the MDGs affecting equity. To identify the effects, it is important to examine through a variety of situations, and variation in child mortality between the poorest and the richest changes as development is made to the MDG. Therefore, the relationship between socioeconomic status and child health has been an area of increasing interest [22–24]. Since the socio-economic variables are related to poverty, the following variables have been included in the study. The explanatory variables were the socio-economic status, demographic and geographic variables including year of survey, age of the women, age at first birth, age at first sex, place of residence, highest educational level of the mother, religion, family size, number of under-five children, number of children ever born, current pregnancy status, birth in the last 5 years, types of birth (single or twin or multiple births), gender of the child, toilet facility, type of fuel, floor material, roof material and wall material. The unit of analysis for this study was considered to be children.

Cox proportional hazard regression models are useful to access the effects of the life-time associated factors on the hazard function. These models have a significant role in the study of the life-time data. In the model, the continuous random variable represents the lifetime of an individual (*t*), and the vector of explanatory variables related to (*X*), when *X* is given under the proportional hazard hypothesis. Therefore, for *x*_*1*_, *x*_*2*_, . . . , *x*_*p*_ be the values of *p* covariates X_1_, X_2_, . . . , X_*p*_. According to the Cox regression model, the hazard function is given as follows:

1 $$ h\left(t,X\right) = {h}_0(t)\psi (X) $$

where *ψ*(*X*) = exp(∑_*i* = 1_^*p*^*β*_*i*_*x*_*i*_), *β* = (*β*_1_, *β*_2_, . . . , *β*_*p*_) is a 1 × *p* vector of regression parameters and *h*_0_(*t*) is the baseline hazard function at that time.

The detailed explanation about the Cox proportional hazard regression model is well documented. This information can be accessed from different books and articles [18–21, 25, 26].

In survival analysis, there are different model diagnosis methods. Most of the diagnostic tests are based on various residuals results. The first model diagnosis tool is based on assessing martingale residual. This method is the default model diagnosis method and useful to assess the goodness of predictor variables. The other model diagnosis technique is based on the deviance residual. The deviance residual method uses the normalized transform from martingale residual. This method is useful to identify poorly predicted subjects. The Cox-Snell residual is also the other model diagnosis tool. This method can be used as the same as in parametric models. The plots of the cumulative hazard function of Cox-Snell residuals give a good strategy for checking goodness of fit of the model.

To find the determinants of under-five mortality when several risk factors were taken into account the analyses were performed using the SAS procedure called PROC PHREG. The goodness of fitness of the model to the data was examined before any inference from the result. These model diagnostics techniques include distributional assumptions assessment; influence diagnostics; residuals and outliers diagnostics [26–28]. One important tool for examining the goodness of Cox’s regression model is the martingale residuals.

Among different models, selecting a suitable model is very important in data analysis. For this purpose, for frailty models, AICs perform different model selection results. Therefore, for the data examined, AIC method appeared to be the best tool for model selection. Moreover, choosing the best model helps to handle confounding effects. For this reason, two stage of model building was used. Each of the predictor variables was fitted one at a time. From the model, significant covariates were remained in the model. Additionally, possible interaction effects between the socio-economic, demographic and geographic variables and year of the survey were added. This process is useful to avoid the difficulty of confounding effects.

The *P* value used in this study is the probability of the observed result when the null hypothesis of the investigation is true. The *p*-value can also be expressed in terms of rejecting the null hypothesis when it is essentially true. In general, the *P*-value can be used as another way to rejection points to give the smallest level of significance for the null hypothesis to be rejected. If the *P*-value is small, it shows stronger evidence is in favour of the alternative hypothesis. Therefore, *P*-values can be obtained from using *p*-value tables, or using different statistical software or spreadsheets.

## Result

Child mortality refers to the infant death and under-five children. From 2000, 2005 to 2011 Ethiopian Demographic and Health Survey (EDHS), it was found that the infant mortality rate, the child mortality rate, and the under-five mortality rate shows a decline. For 2011, the infant mortality rate, the child mortality rate, and the under-five mortality rate was found to be 59 per 1000 live births, 31 per 1000 children surviving to age 1 year and 88 per 1000 live births respectively. Similarly, one in 11 Ethiopian children dies before the fifth birthday. Furthermore, for the 5-year period, neonatal mortality was 37 deaths per 1000 live births, and post-neonatal mortality was 22 deaths per 1000 live births.

Before describing the model results, it is of interest to present the percentage of child mortality in relation to socio-economic, demographic and geographic categorical variables. The under-five children survival rate differs among the three surveys (*P* < 0.0001). Likewise, the under-five children survival/mortality rate differs by region (*p* < 0.0001) with Addis Ababa (92.9 %), the highest survival rate followed by Harari (87.9 %) and Tigray (87.1 %). It is important to note that unlike all other administrative regions, Addis Ababa and Harari are autonomous metropolitans.

Among all the variables presented in Table 1, the under-five children mortality/survival rate difference by the mother’s highest education level or by the mother’s current pregnancy status was statistically insignificant (p > 0.058). As these results are descriptive they give some highlights on how each risk factor is independently associated with the under-five mortality/survival rate. However, several other risk factors must be taken into account when considering the potential effect of a risk factor. The next step is to find the determinants of under-five mortality using the chosen model. The model diagnosis was performed in the analysis. The result is presented in the Additional file 1.

**Table 1:** Distribution between child mortality and socio-economic, demographic and geographic variables

Variables		Survived under-five children					
		Yes	%	No	%	Total	*P*-value
Year of the survey	2000	9501	87.9 %	1307	12.1 %	10,808	<0.000
	2005	8871	91.2 %	851	8.8 %	9722	
	2011	36,791	83.7 %	7179	16.3 %	43,970	
Region	Tigray	6083	87.1 %	902	12.9 %	6985	
	Affar	4443	83.0 %	913	17.0 %	5356	<0.001
	Amhara	7766	84.4 %	1436	15.6 %	9202	
	Oromiya	9027	86.0 %	1473	14.0 %	10,500	
	Somali	3498	86.5 %	546	13.5 %	4044	
	Ben-Gumz	4375	81.8 %	974	18.2 %	5349	
	SNNP	8235	85.3 %	1421	14.7 %	9656	
	Gambela	3299	84.6 %	599	15.4 %	3898	
	Harari	3110	87.9 %	427	12.1 %	3537	
	Addis	2474	92.9 %	190	7.1 %	2664	
	Dire Dawa	2853	86.2 %	456	13.8 %	3309	
Type of place of residence	Urban	8851	89.6 %	1028	10.4 %	9879	<0.001
	Rural	46,312	84.8 %	8309	15.2 %	54,621	
Highest educational level	No education	41,987	84.3 %	7802	15.7 %	49,789	
	Primary	10,300	88.4 %	1356	11.6 %	11,656	0.058
	Secondary	2239	94.0 %	142	6.0 %	2381	
	Higher	637	94.5 %	37	5.5 %	674	
Religion	Christian	31,224	86.3 %	4947	13.7 %	36,171	
	Muslim	22,646	84.5 %	4139	15.5 %	26,785	<0.001
	Other	1275	83.7 %	249	16.3 %	1524	
Currently pregnant	No or unsure	50,077	85.7 %	8356	14.3 %	58,433	0.067
	Yes	5086	83.8 %	981	16.2 %	6067	
Child is twin	Single birth	54,219	86.2 %	8714	13.8 %	62,933	
	1st of multiple	467	59.8 %	314	40.2 %	781	<0.001
	2nd of multiple	476	60.9 %	305	39.1 %	781	
Sex of child	Male	27,960	84.4 %	5158	15.6 %	33,118	<0.001
	Female	27,203	86.7 %	4179	13.3 %	31,382	
Type of toilet facility	No facility	31,730	85.1 %	5566	14.9 %	37,296	
	Flush toilet	1247	89.3 %	149	10.7 %	1396	<0.001
	Pit Latrine	21,095	86.0 %	3431	14.0 %	24,526	
Main floor material	Natural floor/dung	47,253	84.8 %	8457	15.2 %	55,710	
	Wood/parquet	3111	90.7 %	319	9.3 %	3430	<0.001
	Cement	3525	91.2 %	342	8.8 %	3867	
Main wall material	Natural walls	7562	83.2 %	1530	16.8 %	9092	
	Rudimentary walls	34,017	85.0 %	5992	15.0 %	40,009	<0.001
	Finished walls	3111	89.5 %	366	10.5 %	3477	
Main roof material	Natural roofing	24,820	83.8 %	4786	16.2 %	29,606	<0.001
	Rudimentary roofing	9076	86.2 %	1455	13.8 %	10,531	
	Finished roofing	19,446	87.4 %	2810	12.6 %	22,256	

Table 2 gives estimates with hazard ratios for socio-economic, demographic and geographic factors. Based on the findings, there is a variation for the current age of mothers within the EDHS year. As the age of mother’s increased, the hazard ratios were increasing for EDHS years 2000 and 2005. This shows that as the mother’s age increases the risk of the child dying before reaching age 5 increases. But in the 2011 EDHS, the result shows the decrease of under-five mortality risk as the mother’s age increases. The comparisons between the year of the survey show the difference between any two survey years is significant and in recent surveys the effect of the mother’s age weakens. Based on the hazard ratio, the gap between the year 2000 and 2005 is wide. This shows that child mortality was significantly more for 2000 EDHS. But the gap between the 2005 and 2011 EDHSs is small.

**Table 2:** Estimate of the parameters from the proportional Cox Hazard Model

Predictors	Hazard ratio			Hazard ratios difference					
				2000 VS 2011		2005 Vs 2011		2000 Vs 2005	
	2000	2005	2011	95 % CI		95 % CI		95 % CI	
				Lower	Upper	Lower	Upper	Lower	Upper
Current age of respondent	2.95*	1.01*	0.95*	1.32	2.99**	0.02	0.61**	1.77	1.97**
Age at first birth	1.15*	1.16*	1.05*	0.26	0.77**	0.07	0.28**	0.96	1.00
Age at first sex	1.63	1.01	0.89*	0.75	1.05	0.16	0.42	0.73	0.98
Region (Ref. Oromiya)									
Tigray	5.10	4.96	1.17	1.88	4.26**	3.12	5.05**	0.08	0.77
Affar	2.54	2.58	0.82*	0.74	2.67	1.08	2.63**	0.80	1.74**
Amhara	2.88*	2.34*	1.03	1.25	2.96**	0.95	2.08	0.23	0.87**
Somali	1.97	2.46	0.78*	1.29	2.02**	1.03	2.75**	1.20	2.70
Benishangul-Gumz	2.70	2.20	0.93	1.32	3.41**	1.08	3.45**	0.18	0.58**
SNNP	2.79	2.37	0.92	1.92	2.68	1.05	2.81**	0.26	0.89**
Gambela	2.76	2.24	1.07	1.02	2.53**	1.67	2.83	0.41	1.12
Harari	3.22	2.49	1.13	1.46	3.51**	1.14	2.82**	0.62	0.92**
Addis Ababa	0.96	0.76	0.70	0.17	0.72**	0.04	0.52**	0.07	0.56**
Dire Dawa	2.87	2.51	0.91	0.91	1.24	1.11	1.51**	0.23	0.62**
Place of residence (Ref. Urban)									
Rural	1.05	0.98	1.19*	0.68	1.12	0.14	0.69**	0.03	0.69**
Highest educational level (Ref. Higher)									
No education	2.85*	2.31*	0.79*	2.03	3.08**	1.13	1.91**	0.36	0.92**
Primary	2.91	2.53*	0.82*	2.05	2.85**	0.94	1.42	0.15	0.71**
Secondary	2.93*	2.85	0.87*	2.00	2.78**	1.27	2.07**	0.17	0.86
Religion (Ref. Orthodox)									
Muslim	1.76	1.06	1.10*	0.37	0.68**	0.18	0.57	0.21	0.58
Catholic	1.85	1.21	1.01*	1.06	1.39	0.40	0.62	0.15	1.19
Protestant	1.76	0.96	1.09	1.12	1.48	0.26	0.70	0.19	1.34
Traditional	1.55	1.07	0.88	1.15	1.53	0.17	0.79	0.20	0.89**
Other	1.52	1.79	1.71*	0.99	1.44	0.23	0.66	0.19	0.92**
Family size	3.93	1.35	0.99*	2.02	3.84**	0.18	0.88**	1.20	2.28
No of Under five children	1.53	0.99	1.68*	0.54	0.99**	0.58	1.21	0.31	0.88**
Children ever born	1.07*	1.04	1.06*	0.01	0.15**	0.29	0.87	0.07	0.78
Birth in the last five years	3.83*	1.32	1.03	2.00	3.96**	0.17	0.38	1.38	2.73**
Currently pregnant (Ref. No)									
Yes	0.98	1.03	1.06	0.14	0.82	0.09	0.21	0.03	0.65
Type of birth (Ref. Single birth)									
1st of multiple	0.56*	0.79	1.18	0.52	1.43	0.35	0.91**	0.81	0.85
2nd of multiple	1.05	1.14	2.00	0.50	1.56	0.15	0.79	0.04	0.56**
3rd of multiple	1.39	1.38*	0.56*	0.48	0.85**	0.74	1.10	0.06	0.25
Sex of the child (Ref. Female)									
Male	1.08	1.19	1.09*	0.01	0.34**	0.04	0.33**	0.07	0.28**
Toilet facility (Ref. Pit latrine)									
Flush toilet	3.92	0.73	1.13	0.37	1.94	0.59	1.27	2.03	3.10
No facility	1.02	0.92	1.01	0.48	1.98	0.16	2.01	0.63	0.72
Type of fuel (Ref. Charcoal/wood etc.)									
Electricity	1.10	2.19	2.16*	0.77	1.23	0.24	0.84	0.81	1.22
Gas/LPG	0.43*	0.39*	1.35	0.44	0.84	0.40	0.89	0.01	0.37**
No food cooked	1.13	1.01	0.63	0.99	1.09	0.35	1.12	0.18	0.74
Floor material (Ref. Carpet)									
Cement	1.05	0.73	1.12	0.20	1.43	0.30	2.06	0.65	1.24
Natural floor	1.15	0.92	0.94	0.03	1.93	0.95	2.37	0.57	1.17
Wood/ceramics	1.56*	0.63	0.77	0.83	1.02	0.62	1.09	0.49	1.10
Roof material (Ref. Thatch/Plastic/wood)									
Finished roofing	1.15	0.93	1.00	0.69	1.04	0.73	1.42	0.28	0.83
No roof	1.56	0.95	0.99	0.04	1.99	0.20	1.72	0.74	1.26
Wall material (Ref. Finished walls)									
No walls	1.06	0.93	0.82	0.98	1.33	0.58	1.28	0.25	0.69
Cane/bamboo/wood	0.99	1.04	0.81*	0.80	1.96	0.92	1.80	0.11	0.25

* Significant at 5 % level of significance

**Significant interaction effects

The age at 1^st^ birth was significant in all the EDHS years. Notably, the effect of age at 1^st^ birth reduces in the recent surveys. The result is presented in Table 2. The gap between the years 2000 and 2005 is very small. But after the 2005 survey, the result shows the declining trend in under-five mortality in the year 2011 for age at first birth. On the other hand the rural-urban difference is insignificant between the three EDHS years, though the 2011 result of EDHS only shows a significant difference between rural and urban under-five mortality hazard ratios (Table 2).

**Fig. 1:**
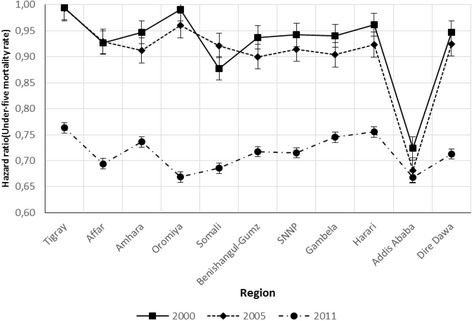
Hazard ratio associated with under-five mortality and region with year of the survey

The other significant effect is the joint effect of region and year of the survey (Table 2). Therefore, from the analysis, it can be identified that child mortality is higher for the Tigray region for 2000 and for 2005 followed by Oromiya region for 2000 and for 2005. Moreover, the trend of child mortality displays a decreasing trend for the year 2011 compared to years 2000 and 2005 for all regions (Fig. 1).

**Fig. 2:**
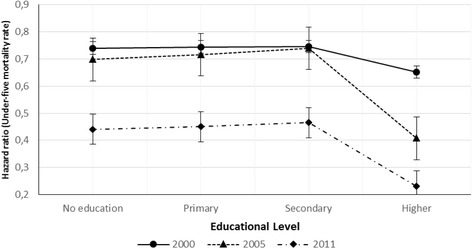
Hazard ratio associated with under-five mortality and educational level with year of the survey

The other significant effect is the between educational level and survey year. Likewise, the year of reference is 2011. Therefore, the output shows that mothers with no education have a lower chance for the child to be dead before reaching age five for all EDHS survey years followed by mothers with primary and secondary education (Table 2). When compared between years, the under-five mortality is significantly higher for the year 2000 followed by 2005 and 2011 (Fig. 2).

On the other hand, Table 2 shows that there is significant effect between EDHS surveys and family size. As the analysis shows, for the year 2000, child mortality increased by 2.93 % for an increase in a number of household members in the house in comparison to the year 2011. Likewise, for the year 2005, child mortality is higher by 0.35 % for an increase in a number of household members in the house compared to the year 2011. Furthermore, the under-five mortality is lower as the year of study increases (2000, 2005 and 2011) and the child mortality is higher for an increase in family size. For households with more under-five children in the house, under-five mortality shows an increase for the years 2000 and 2011. But for the year 2005, the analysis gives that child mortality shows a decrease for an increase in the number of under-five children in the household (Table 2).

**Fig. 3:**
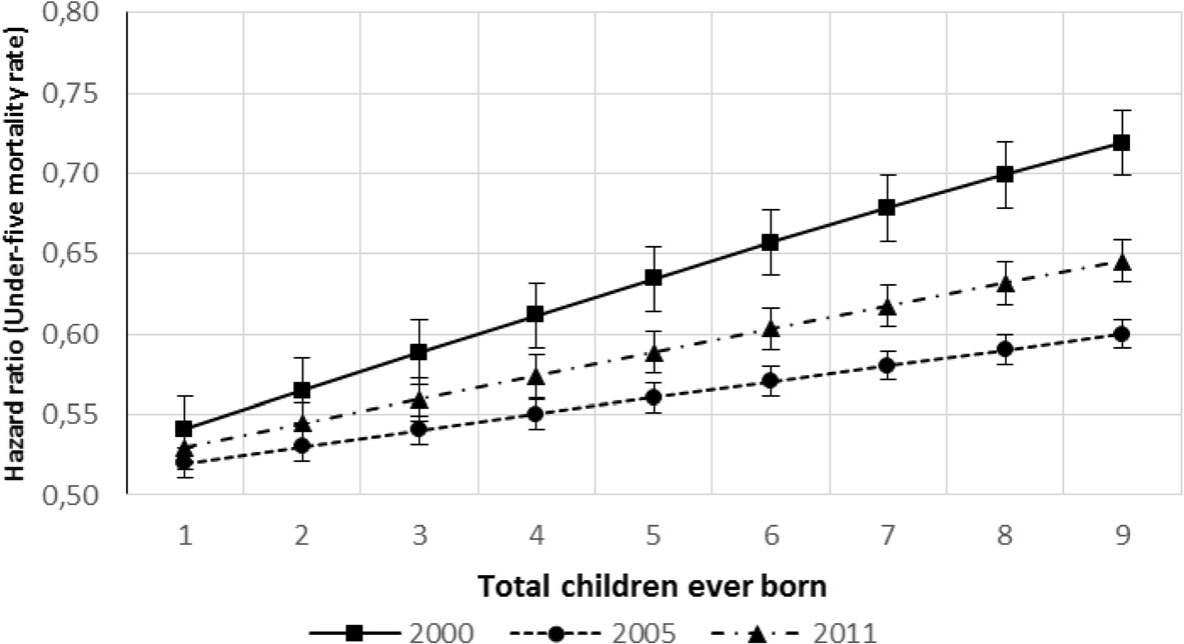
Hazard ratio associated with under-five mortality and total children ever born in the house with year of the survey

Furthermore, Table 2 presents the significant factors of child mortality and EDHS surveys. As the result indicates, the effect of total children ever born is not similar between the survey years. The occurrence for the child not to be alive before celebrating age five is higher for the year 2000 followed by the year 2011. EDHS 2005 survey shows a decline in under-five mortality compared to 2000 and 2011. As can be seen in Fig. 3, under-five mortality increases as the number of children ever born increases for all EDHS survey years.

**Fig. 4:**
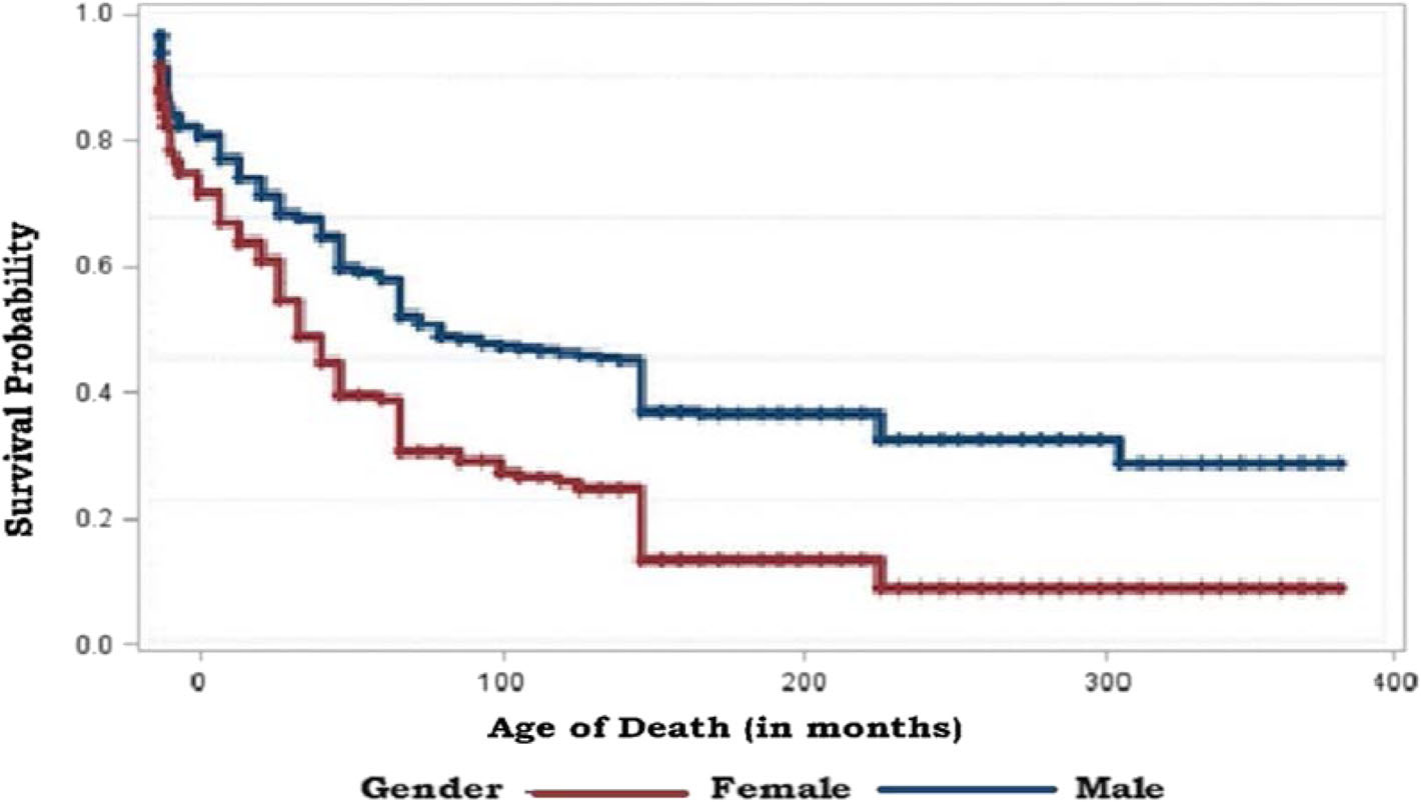
Survival function at mean of covariates

The estimated survival curve for children under the age of five for male and female is given in Fig. 4. From the figure, the survival curves give a visual representation of the life tables. From the figure, it can be seen that male children were at a higher risk of death than female children. The equality of the survival function among groups can be tested using non-parametric methods (Fig. 4). Figure 4 of the estimator stratified by gender below shows that females generally have a worse survival experience. This is supported by the three significant tests of equality. These are Log-Rank (Chi-square = 7.7911, *P*-value =0.0053), Wilcoxon (Chi-square = 5.5370, *P*-value = 0.0186) and -2Log (LR) (Chi-square = 10.5120, *P*-value = 0.0012). Moreover, as the age of a child increases the probability of the child to survive is decreasing for both male and female and at the end, the survival for both male and female is the same.

## Discussion

In this research, the trends of under-five mortality with the effects of socio-economic, demographic and geographic in Ethiopia using 2000, 2005 and 2011 EDHS was investigated. For this study, the nationally representative data from 2000, 2005 to 2011 Ethiopian Demographic and Health Surveys was used. The Cox regression analysis technique has been useful to find the significant socio-economic, demographic and geographic covariates of under-five mortality over the years. Cox regression model can be used when the relative risk values for different levels of variables are measured at different levels of socio-economic, demographic and geographic variables. A study which involves an outcome variable of interest with the time to the occurrence of an event can be modelled using the proportional hazards model. For this kind of situation using logistics regression method ignores the actual failure time and models the occurrence or non-occurrence of the event. But the inclusion of the time to event information is important to obtain the actual and accurate estimates [18]. Even though infant and child mortality rates have been decreasing in many parts of sub-Saharan Africa in recent decades, it remains among the highest in the world. In literature, the decrease in mortality is recorded in detail. From the documentation, it was not possible to identify the several socio-economic, demographic and geographic factors. Moreover, identifying the pattern of mortality by means of different years of surveys is essential.

Therefore, for this study, the Cox Regression Method was used. The Cox Regression procedure is useful for modelling the time to a specified event, based on the values of given covariates. Several socio-economic, demographic and geographic predictors were used to estimate the level of under-five mortality to find trends. Moreover, the death of a child (status variable) is binary and dependent variable in Cox regression. Time variable (age at death) measures the extent to the event defined by the status variable. The covariates (independent variables) for this study contain both categorical and continuous variables.

The findings of this study which incorporates the child mortality in Ethiopia displayed that socio-economic, demographic and social factors have a great effect on the child mortality between the years of 2000, 2005 and 2011. These factors are age at 1st birth, place of residence, educational level, family size, number of children under five, total children ever born and age of respondents. The result from the study suggests that one in 17 Ethiopian children dies before their first birthday. Furthermore, the Cox regression model determined that the data obtained during 2000, 2005 and 2011 Ethiopian Demographic and Health Survey has a great importance and it captures method-dependent.

On the basis of this study, child mortality is greater in 2000 followed by 2005 in comparison to 2011. Furthermore, the risk of under-five mortality declines from 2000 to 2011 for educational level. In the same way, number of household members and the number of under-five children in the house display differences between years. From the analysis, the age of the women displays differences between years. Consequently, child mortality is declining from 2000 to 2011. For a growth in total children ever born the possibility of a child to die is greater for the year 2000 followed by 2005 and 2011. But for the number of last 5 years birth, child mortality increased from 2000 to 2011. Therefore, for the women having more children in 5 years has to be avoided to reduce the risk of under-five mortality.

## Conclusion

Finally, for educated mothers giving birth at the older age have higher chances for a child to reach age five. Furthermore, for unemployed mothers living in households with large members, under-five mortality is higher. Consequently, for women who are not pregnant, giving birth at older ages reduces child mortality. In addition to this child mortality varies by regions for the three survey years. But, in some regions, the decline for child mortality between years was observed. To reduce child mortality, implementing better education for family planning and child health care is an important strategy which has to be implemented by the government. For further study, the structured additive model which incorporates the spatial variation between primary sampling units can be used to assess the effect of socio-economic, demographic and geographic factors.

The limitation of EDHS survey can be associated with the following criticisms. These include the EDHS data only collected from women aged between the ages of 15 and 49 who are alive in a given household. But for mothers who have died, no data was collected. This situation can create a bias in the results. The technique used in the EDHS survey is a retrospective method. This is related to various difficulties and challenges. The main difficulty with this challenge is related to the quality of the results because it depends on the completeness and correctness of the birth and death history. Therefore, the conclusions and recommendations of the study can be affected by this problem. The other limitation is related to the sample size. The sample size is the only representative at the highest administrative unit of the country. Therefore, the dataset cannot be used to estimates at the lowest administrative units.

## Additional file

Additional file 1: Figure A1. A) Martingale Residual Plot and B) Deviance Residual Plot. Figure A2. Cumulative Martingale residuals for age, age of respondents at 1st birth, number of household members and total children ever born (DOCX 156 kb)

## Abbreviations

AIC: Akaike information criterion
DHS: Demographic and Health Survey
EDHS: Ethiopian Demographic and Health Survey
