# Supplementary material for: Comparison of under-five mortality for 2000, 2005 and 2011 surveys in Ethiopia
Source: BMC Public Health. 2016 Sep 5;16(1):930. doi: 10.1186/s12889-016-3601-0 (PMC5011871; doi:10.1186/s12889-016-3601-0)
Supplement: Additional file 1: Figure A1. — A) Martingale Residual Plot and B) Deviance Residual Plot. Figure A2. Cumulative Martingale residuals for age, age of respondents at 1st birth, number of household members and total children ever born (DOCX 156 kb) [file 12889_2016_3601_MOESM1_ESM.docx]

**Appendix**

The model diagnosis was performed in the analysis. From the result, the maringate residuals are skewed because of the single event setting of the Cox model. The martingale residual plot in Figure A1.A shows an isolation point, but this observation is no longer distinguishable in the deviance residual plot in Figure A1.B. These plots indicate the goodness of fit of the model to individual observations. Moreover, Figure A1.A shows the grouped martingale residual processes when the individuals are aggregated over groups. Figure A2 displays the observed cumulative martingale residuals for the covariates; namely mother’s age, the age of the mother at 1st birth, household size and the total number of children the mother ever born together with simulated realizations from the null distribution. The observed process cumulative martingale residuals compared to the simulated realizations provided the goodness of the relative risk regression models based on the three EDHS data set.

| 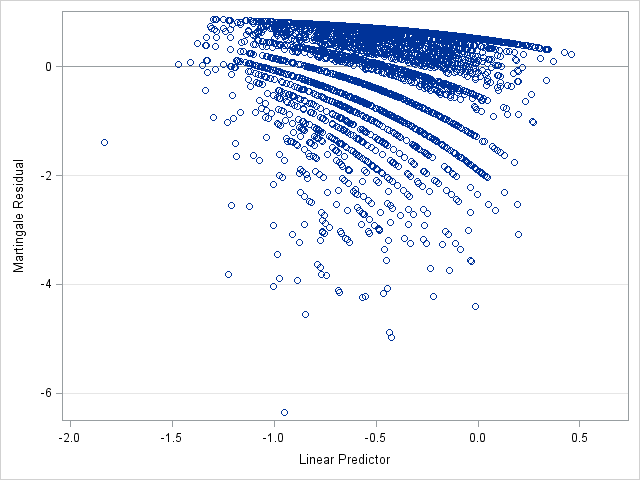 |  | 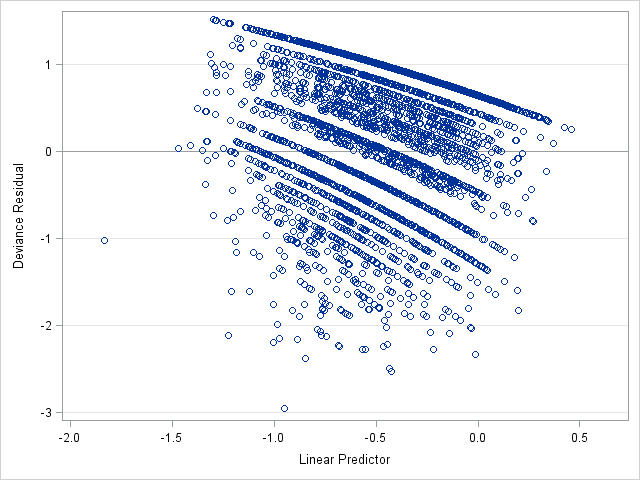 |
| --- | --- | --- |
| A |  | B |

**Figure A1. A) Martingale Residual Plot and B) Deviance Residual Plot**

| 1. 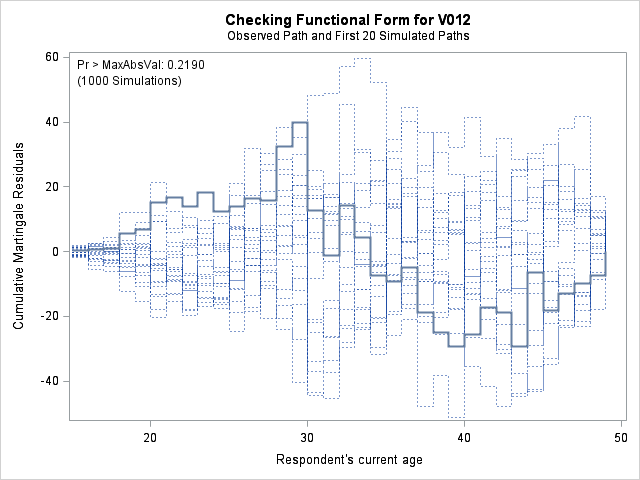Respondent’s age |  | 1. 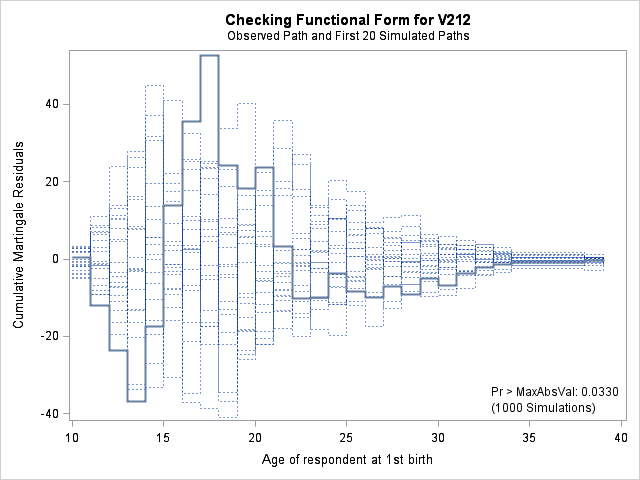Age of respondents at 1^st^ birth |
| --- | --- | --- |
| 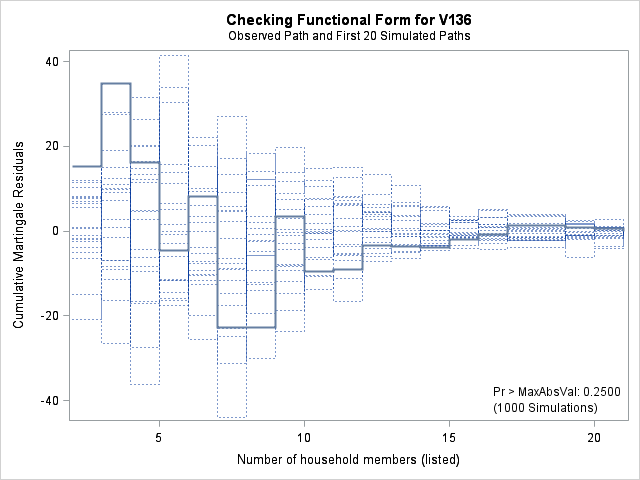 |  | 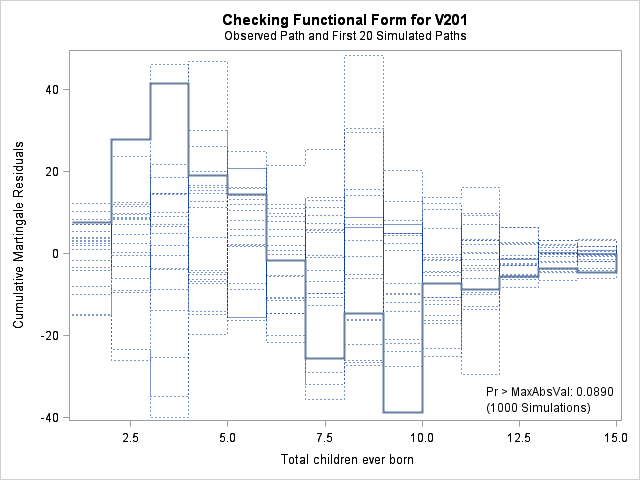 |
| C) Number of household members |  | D) Total children ever born |

**Figure A2. Cumulative Martingale residuals for age, age of respondents at 1st birth, number of household members and total children ever born**
